# Bonding Pictures: Affective Ratings Are Specifically Associated to Loneliness But Not to Empathy

**DOI:** 10.3389/fpsyg.2017.01136

**Published:** 2017-07-10

**Authors:** Heraldo D. Silva, Rafaela R. Campagnoli, Bruna Eugênia F. Mota, Cássia Regina V. Araújo, Roberta Sônia R. Álvares, Izabela Mocaiber, Vanessa Rocha-Rego, Eliane Volchan, Gabriela G. L. Souza

**Affiliations:** ^1^Department of Biological Sciences, Federal University of Ouro PretoOuro Preto, Brazil; ^2^Institute of Biophysics Carlos Chagas Filho, Federal University of Rio de JaneiroRio de Janeiro, Brazil; ^3^Center for the Study of Emotion & Attention, University of Florida, GainesvilleFL, United States; ^4^Department of Natural Sciences, Institute of Humanities and Health, Federal Fluminense UniversityRio das Ostras, Brazil

**Keywords:** social interaction, bonding, arousal, valence, IAPS, empathy, loneliness

## Abstract

Responding to pro-social cues plays an important adaptive role in humans. Our aims were (i) to create a catalog of bonding and matched-control pictures to compare the emotional reports of valence and arousal with the International Affective Picture System (IAPS) pictures; (ii) to verify sex influence on the valence and arousal of bonding and matched-control pictures; (iii) to investigate if empathy and loneliness traits exert a specific influence on emotional reports for the bonding pictures. To provide a finer tool for social interaction studies, the present work defined two new sets of pictures consisting of “interacting dyads” (Bonding: *N* = 70) and matched controls “non-interacting dyads” (Controls: *N* = 70). The dyads could be either a child and an adult, or two children. Participants (*N* = 283, 182 women) were divided in 10 groups for the experimental sessions. The task was to rate the hedonic valence and emotional arousal of bonding and controls; and of pleasant, neutral, and unpleasant pictures from the IAPS. Effects of social-related traits, empathy and loneliness, on affective ratings were tested. Participants rated bonding pictures as more pleasant and arousing than control ones. Ratings did not differentiate bonding from IAPS pleasant pictures. Control pictures showed lower ratings than pleasant but higher ratings than neutral IAPS pictures. Women rated bonding and control pictures as more positive than men. There was no sex difference for arousal ratings. High empathic participants rated bonding and control pictures higher than low empathic participants. Also, they rated pleasant IAPS pictures more positive and arousing; and unpleasant pictures more negative and arousing than the less empathic ones. Loneliness trait, on the other hand, affected very specifically the ratings of bonding pictures; lonelier participants rated them less pleasant and less arousing than less lonely. Loneliness trait did not modulate ratings of other categories. In conclusion, high empathy seems related to emotional strength in general, while high loneliness seems to weaken the engagement in social interaction cues.

## Introduction

Previous studies have shown that affective pictures drive the activity of brain networks and impact behavior (Pereira et al., 2006, 2010; Sanchez et al., 2015). A catalog with 100s of emotional and neutral pictures, the International Affective Picture System (IAPS) (Lang et al., 2005), and a scale, the Self-Assessment Manikin (SAM), were developed to evaluate the hedonic valence (pleasantness/unpleasantness) and the emotional arousal evoked when viewing these pictures (Bradley and Lang, 1994). Studies have proposed that emotional reactions are organized around two motivational states — appetitive and defensive — that have evolved to promote the survival of species. Exposure to pictures displaying erotic scenes, nature, families, food and sports would promote activation of appetitive systems; while exposure to those depicting threat scenes and mutilations would promote activation of defensive systems (Bradley et al., 2001a).

One of the most important characteristics of human beings is their social nature. The successful establishment of a social group depends largely on the capacity of the individuals of a species to recognize one another and interpret the emotional states of other members of the group accurately (Öhman, 2006). It has been shown that humans are very proficient in detecting social interaction cues which encourage social bonding (Over and Carpenter, 2009; Centelles et al., 2011; Campagnoli et al., 2015). More specifically, some studies have indicated that affiliative stimuli (e.g., pictures of babies and families) activate motor cerebral circuits “prepared” for social interaction (Brosch et al., 2007; Caria et al., 2012; Souza et al., 2012; Campagnoli et al., 2015). Souza et al. (2012) showed that exposure to pictures of babies and families facilitate finger flexion – a movement that could be analogous to social touching or to grooming behavior in animals. Campagnoli et al. (2015), presenting pictures of social bonding (dyads of a child and an adult or a child and a child) and employing electroencephalography recordings to analyze the motor readiness potential, suggested that social interaction stimuli prepare individuals to interact with each other by activating pre-existing motor circuits for actions (fingers flexion) compatible with caressing (e.g., gently stroking a very soft cloth).

Some social traits could positively or negatively influence the recognition and evaluation of social stimuli and, consequently, boost or harm the formation and quality of social networks, promoting or undermining health. Empathy is a complex phenomenon and it is considered to be related to social function. Despite the lack of a clear definition, there is a consensus that empathy involves at least three different processes: feeling what another person is feeling, knowing what another person is feeling, and having the intention to respond compassionately to another person’s distress (Levenson, 1996). The present work will address the “feeling what another person is feeling” definition for empathy. This process has great importance in social life, since it enables an individual to more accurately predict the needs and actions of other people (de Vignemont and Singer, 2006). There is considerable evidence suggesting that empathy has a deep evolutionary, neuroendocrinal and neurophysiological base (Decety et al., 2012), and has been fundamental in human development. Empathy allows humans to understand quickly and automatically other people’s emotions, which facilitates more successful social interactions by helping friendship reinforcement, reciprocity and self-interest (Batson and Ahmad, 2001).

Loneliness is one of the main indicators used when assessing well-being. Loneliness stems mainly from the way people perceive, evaluate and respond to interpersonal situations, and the perspective of being alone does not result from the number of contacts, but from their quality (Perlman and Peplau, 1981; Hawkley et al., 2008). Cacioppo and Cacioppo (2012) argue that people who subjectively feel they are isolated or have few, if any, strong connections to others live less than those who feel they have strong and meaningful social bonds. Moreover, important reviews regarding prospective epidemiological studies on social isolation among humans have shown that this situation is a risk factor of morbidity and mortality as strong as smoking, obesity, sedentarism, and hypertension (House et al., 1988; Holt-Lunstad et al., 2010). Longitudinal studies have demonstrated that perceived social isolation in humans has many aspects in common with the effects of experimental isolation on social animal species, such as increased sympathetic tonus, activation of the hypothalamic-pituitary-adrenal axis, decreased inflammatory control, immune deficit and sleeping problems, as well as expression of genes regulating the glucocorticoid response and resistance (Cacioppo et al., 2006; Cacioppo and Hawkley, 2009). Interestingly, it has been shown that some neural regions processing basic life-threatening signals are also involved in the processing of information on threats to social bonds (Eisenberger, 2013). On the other hand, establishing strong social bonds is expected to result in positive health outcomes.

To provide a finer tool for social interaction studies, the present work defined two new sets of pictures consisting of bonding scenes depicting “interacting dyads” and matched controls depicting “non-interacting dyads.” Special features of the experimental and control pictures are (a) each pair is portrayed by the same individuals, (b) the pair is photographed with the same background, and (c) all dyads have at least one child. These features ensure that interaction would be the only factor differentiating the bonding and control pairs. Our aims were (i) to use the new catalog of bonding and matched-control pictures to compare the emotional reports of valence and arousal with the IAPS pictures; (ii) to verify sex influence on the valence and arousal of bonding and matched-control pictures; (iii) to investigate if empathy and loneliness traits exert a specific influence on emotional reports for the bonding pictures.

## Materials and Methods

### Participants

Two hundred eighty three undergraduate students (182 women and 101 men) aged between 18 and 35 years old (mean = 21.5 years, *SD* = 2.90) comprised the study sample. Participants were from different courses of the Federal University of Ouro Preto (Brazil). This study was carried out in accordance with the recommendations of the Ethics Institutional Review Board of the Federal University of Ouro Preto with written informed consent from all subjects. The protocol was approved by the Ethics Institutional Review Board of the Federal University of Ouro Preto (CAAE: 32885314.2.0000.5150).

### Visual Stimuli

**FIGURE 1:**
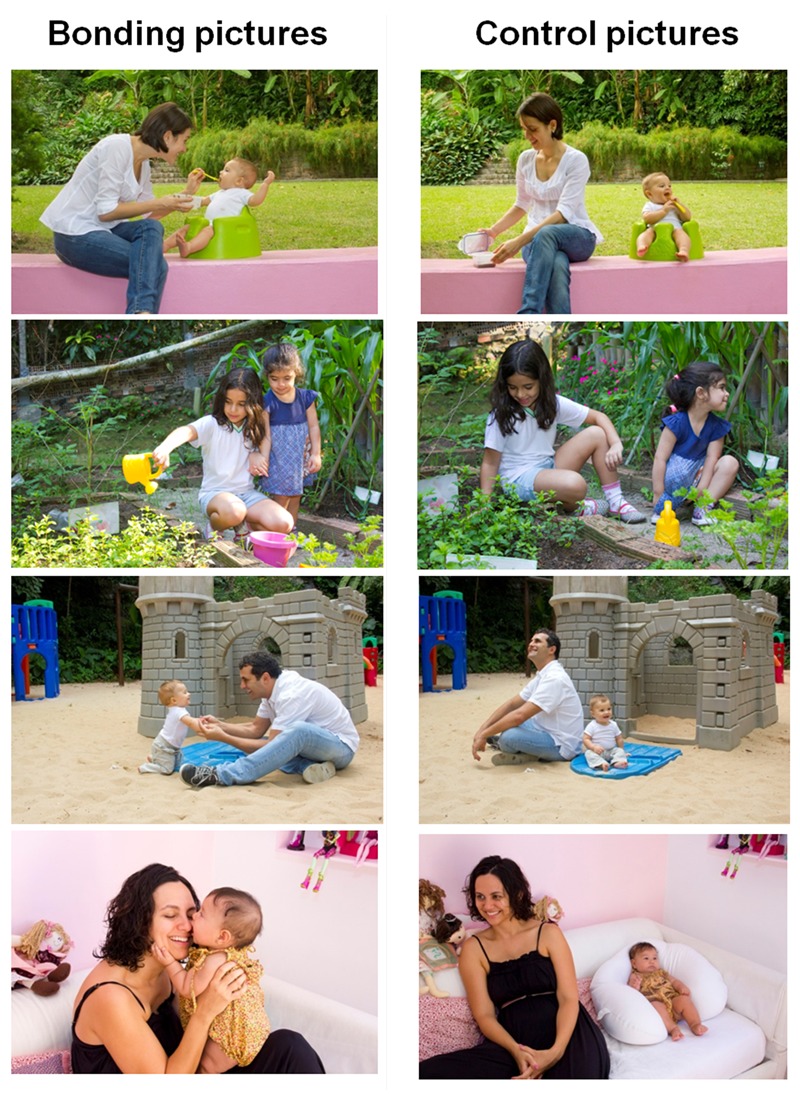
Examples of the new set of pictures: bonding pictures **(left)** and control pictures **(right)**.

Pairs of bonding and control pictures were taken by a professional photographer providing 140 pictures (70 bonding pictures and 70 matched control pictures). Pictures always comprised two individuals, being one of them a baby or a child. The other person on the picture could be either another baby/child or an adult. In the bonding pictures, the dyads could be interacting through visual contact (16 pictures), social touch (12 pictures), visual contact plus social touch (28 pictures), hug (5 pictures), or lap (9 pictures). Each matched control picture depicted the same dyad photographed against the same background (school, garden, playground, etc.). Control pictures involved no-direct social interaction between them; the dyads were back-to-back and/or performing single actions or simply looking at opposite directions. Because the pictures comprise babies and children we are not able to make these available for the scientific community. We do not have the image rights to release the pictures to other researchers. **Figure 1** shows four examples of bonding and control pictures pairs.

Seventy six pictures were selected from the IAPS (Lang et al., 2005): 30 unpleasant (mutilated bodies, accidents, animal attacks, human violence, losses, disease, and pollution), 30 neutral (household items and mushrooms) and 16 pleasant (erotic scenes, adventure, sports and food)^1^. Based on Lang colleagues (Bradley et al., 2001a), valence (mean ± standard deviation) was 7.2 ± 0.38 for pleasant pictures, 5.0 ± 0.31 for neutral pictures and 2.5 ± 0.72 for unpleasant pictures; and arousal (mean ± standard deviation) was 5.4 ± 1.06 for pleasant pictures, 2.8 ± 0.58 for neutral pictures, and 6.0 ± 0.95 for unpleasant pictures.

Within the selected IAPS categories (unpleasant, neutral, and pleasant), the contents of the scenes were fairly heterogeneous, whereas the current named bonding and matched-control categories are uniform. Bonding and control pictures depict dyads which are either interacting (bonding) or not interacting (control). Additionally, each bonding and matched control pair depicts a same dyad embedded in the same background scene.

### Evaluative Reports

The pictures were evaluated using the paper-and-pencil version of the SAM (Bradley and Lang, 1994), which consists of pictorial drawings of manikins representing the dimensions of hedonic valence and emotional arousal. For each dimension, there is a row of five figures interleaved by blank spaces, yielding nine intensity levels. For the hedonic valence dimension, the manikins exhibit expressions that range from “smiling-happy” (score = 9) to “frowning-unhappy” (score = 1). For the emotional arousal dimension, the expressions of the manikins range from an “excited wide-eyed” figure (score = 9) to a “relaxed-sleepy” figure (score = 1).

Ten sessions, each with a different group of participants (ranging from 24 to 33 students), were performed. Every session presented the 76 IAPS pictures to display a “normative” background and serve as comparison for ratings of the bonding and control pictures. Those were divided in 10 different sets of 14 pictures so that each session presented seven bonding and seven control pictures.

### Social-Related Traits

#### Loneliness

The UCLA-revised loneliness scale (Russell et al., 1980), which was translated into and adapted for Portuguese (Neto, 1989), was used for assessing perceived social isolation. The UCLA loneliness scale is an 18-item questionnaire aimed to assess participant’s self-perception of loneliness and the feelings associated with it. In this scale, the options available are “never,” “rarely,” “sometimes,” and “many times” (with the score ranging from 1 to 4 points, respectively), with items 1, 4, 5, 8, 9, 13, 14, 17, and 18 having a reverse score. The internal consistency of the Portuguese version of the UCLA loneliness scale was high with a Cronbach’s alpha of 0.87 (Neto, 1989).

#### Empathy

The emotional contagion scale (Doherty, 1997), translated into and adapted to Portuguese (Gouveia et al., 2007), was used to assess empathy. The emotional contagion scale is also an 18-item questionnaire designed to assess the general ability of individuals to pay attention to the feelings of others or to be affected by them. So, it assesses only one of the three processes outlined above that are usually described to define empathy. In this scale, the options available are “never,” “rarely,” “often,” and “always” (with a score ranging from 1 to 4 points, respectively). The internal consistency of the Portuguese version of the emotional contagion scale was high with a Cronbach’s alpha of 0.82 (Gouveia et al., 2007).

### Apparatus

A microcomputer containing the Microsoft Power Point slides controlled both the order and the timing of the stimuli presentation. Using an Epson projector, the pictures were displayed on a white screen where the stimuli had an average size of 2.0 m (horizontal) and 1.5 m (vertical).

### Procedure

The experiment was conducted in a dimly lit classroom with comfortable desks placed in rows in front of a slide projection screen. The desks were arranged in such a manner that the screen was completely visible to every participant. A didactic video explained the upcoming task, and a practice task was performed using nine IAPS pictures (Bradley et al., 2001a) (three neutral, three unpleasant, and three pleasant)^2^ to allow the participants to learn how to appropriately use the paper-pencil SAM scale (Öhman, 2006) after viewing each image.

Each rating trial began with a preparation slide with the sentence “Look attentively at figure X” (displayed for 3 s), followed by the picture presentation for 6 s. A beep was synchronized with the initial display of the picture, indicating that participants should look at the picture until its offset, when another beep was played. Then a slide showing the sentence “Please rate figure X according to the scales” was presented and, for the next 10 s, participants were instructed to rate the picture along the dimensions of hedonic valence and emotional arousal using the paper and pencil version of the SAM scales (Bradley and Lang, 1994).

Participants rated 90 images. Fourteen out of which belonged to the category of interest: seven bonding and seven control pictures. The remaining 76 pictures were from the IAPS (Lang et al., 2005). The sequential order of the picture presentation was pseudo-randomized with the constraint that specific content could not be repeated more than twice consecutively. At the end of the rating session, participants filled out the empathy and loneliness traits scales. Each experimental session lasted approximately 1 h.

### Statistical Analyses

Data were analyzed using the Statistical 7.0 software program (StatSoft, Inc.). Statistical significance was taken as *p* < 0.05.

Two different types of analyses were performed: (i) analyses where mean ratings from participants were calculated for each picture and (ii) analyses where mean ratings from pictures of a given category were calculated for each participant.

#### Analyses per Picture

The mean and standard-deviation of ratings from the participants of the present study were computed for each picture, separately for the valence and arousal dimensions. To ensure the experiment followed the methodology proposed by Lang et al. (2005) we analyzed the participants’ ratings for unpleasant, neutral and pleasant pictures and compared them with their normative values from the IAPS (Lang et al., 2005) using Pearson correlations applied for comparing both valence and arousal of the pictures.

To assess the consistency of the images’ bi-dimensional distribution, the model proposed by Greenwald et al. (1989) was used. According to this model, valence and arousal ratings of a heterogeneous emotion-laden group of pictures plotted in a Cartesian plan are disposed in vectors that point in two directions, representing a “boomerang” shape. The upper arm of the boomerang indexes appetitive (approach-like) motivation, and the lower arm indexes defensive (avoidance-like) motivation. Further, to check how the ratings for bonding and control pictures relate to the ratings of mixed categories of IAPS unpleasant, neutral and (specially) pleasant pictures, an one-way ANOVA was performed with PICTURES CATEGORY (pleasant, neutral, unpleasant, bonding, and control pictures) for valence and arousal separately. *Post hoc* tests were performed using Newman–Keuls.

To compare each bonding picture to its corresponding control, that is, to the picture portraying the same dyad but in a non-interacting condition we used Student’s *t*-tests for dependent samples separately for valence and arousal.

#### Analyses per Participant

Sex differences in valence and arousal were analyzed with a mixed design ANOVA, with PICTURES (bonding and control) as the within-subject factor and SEX (men and women) as the between-subject factor separately for valence and arousal. *Post hoc* tests were performed with Newman–Keuls.

To test the influence of empathy and loneliness traits on affective ratings of bonding pictures, the sample was divided into low and high sub-groups by the median split. To balance the number of men and women in each sub-group, the median value for women and the median value for men were used for splitting. Low and high empathy sub-groups, and low and high loneliness sub-groups were tested separately. Analyses were performed through repeated measures ANOVAs with PICTURES CATEGORY (bonding and control pictures), as within-subject factor, and EMPATHY TRAIT (low and high), as between-subject factor; for valence and arousal separately. Similarly, analyses were performed through repeated measures ANOVAs with PICTURES CATEGORY (bonding and control pictures) and LONELINESS TRAIT (low and high), for valence and arousal separately.

To investigate whether the influence of traits was specific to social interaction scenes, analyses were also performed on ratings of IAPS pictures. Tests were run through repeated measures ANOVAs with PICTURES CATEGORY (unpleasant, neutral, and pleasant), as within-subject factor, and EMPATHY TRAIT (low and high), as between subject-factor; for valence and arousal separately. Similarly, analyses were performed through repeated measures ANOVAs with PICTURES CATEGORY (unpleasant, neutral, and pleasant) and LONELINESS TRAIT (low and high), for valence and arousal separately.

*Post hoc* tests were performed using Newman–Keuls.

## Results

The average score reported for IAPS pictures in the present study was compared to that reported for North Americans (Lang et al., 2005). There was a positive correlation between the hedonic valence (unpleasant: *r* = 0.83, *p* < 0.001; neutral: *r* = 0.49, *p* = 0.006 and pleasant: *r* = 0.59, *p* = 0.02) and the emotional arousal (unpleasant: *r* = 0.83, *p* < 0.001; neutral: *r* = 0.63, *p* < 0.001; and pleasant: *r* = 0.91, *p* < 0.001).

**FIGURE 2:**
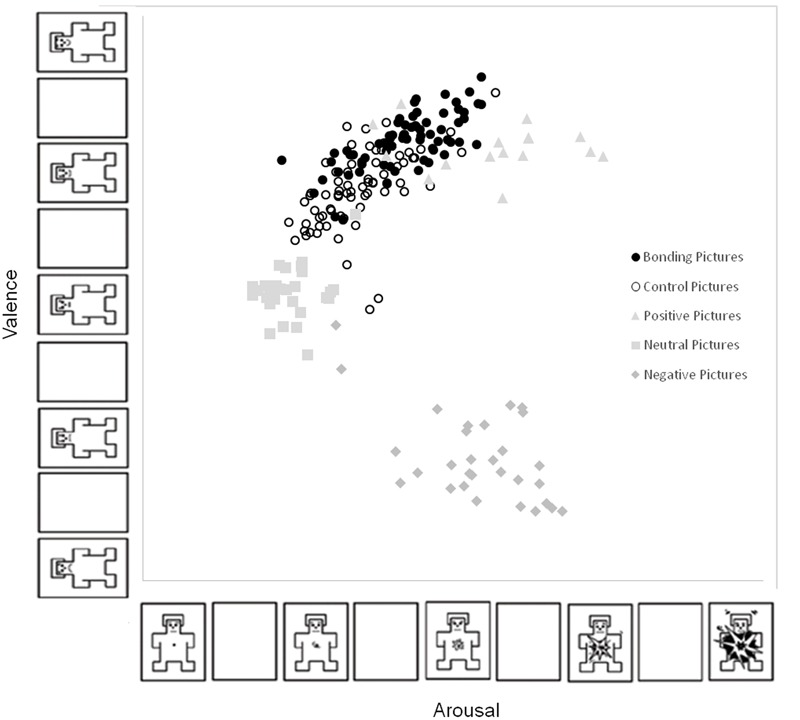
Affective space: bi-dimensional plot of each picture as a function of its mean valence ratings (y axis) and arousal ratings (x axis). Each point in the graph represents the mean for each picture according to all participants’ ratings. Bonding pictures (filled black circle); control pictures (empty black circle), pleasant pictures (gray triangle); neutral pictures (gray square); and unpleasant pictures (gray diamond).

**Figure 2** shows the mean values of valence and arousal in the Cartesian plane regarding pictures of interest for this study (i.e., bonding and control pictures) as well as pleasant, neutral and unpleasant IAPS pictures rated by the present sample. The distribution pattern of IAPS pictures revealed the same typical boomerang shaped distribution as described by Bradley et al. (2001a). Ratings for bonding and control pictures were classified in the “pleasant” domain considering IAPS normative instructions [Valence (mean ± standard deviation) for Bonding: 7.01 ± 0.92 and Control 6.31 ± 1.04; Arousal (mean ± standard deviation) for Bonding: 4.35 ± 0.62 and Control: 3.79 ± 0.62].

The investigation of differences in valence among the new catalog of bonding and matched-control pictures and IAPS pictures showed a main effect of PICTURE CATEGORY (*F*_(4,215)_ = 154.4, *p* < 0.001, $\eta_{p}^{2}$ = 0.95). Participants rated bonding pictures as more positive than all other categories (*p* < 0.001 for all comparisons), except the “pleasant.” Control pictures were rated as more positive than unpleasant and neutral pictures (*p* < 0.001 for both) but less positive than pleasant and bonding pictures (*p* < 0.001 for both). The investigation of differences in arousal among the new catalog of bonding and matched-control pictures and IAPS pictures showed a main effect of PICTURE CATEGORY (*F*_(4,215)_ = 81.15, *p* < 0.001, $\eta_{p}^{2}$ = 0.81). Participants rated bonding pictures as more arousing than neutral and control pictures (*p* < 0.001 for both) but less arousing than pleasant and unpleasant pictures (*p* < 0.001 for both). Control pictures were rated less arousing than all other categories (*p* < 0.001 for all) except the “neutral” (*p* < 0.001). As expected, valence ratings for bonding pictures did not significantly differ from valence ratings for the mixed set of pleasant pictures. Not unexpected, arousal ratings for the mixed set of pleasant pictures, which included high arousing erotic and adventures scenes, were higher than arousal ratings for bonding pictures.

Compared to their matched controls, bonding pictures were rated as being more pleasant (*t* = 4.37; *p* < 0.0001) and more arousing (*t* = 5.64; *p* < 0.0001).

The investigation of differences of sex between the valence of bonding and control pictures showed a main effect of PICTURE CATEGORY (*F*_(1,260)_ = 192.52, *p* < 0.001, $\eta_{p}^{2}$ = 0.43), a main effect of SEX (*F*_(1,260)_ = 14.76, *p* < 0.001, $\eta_{p}^{2}$ = 0.05), and an interaction between PICTURE CATEGORY and SEX (*F*_(1,260)_ = 9.79, *p =* 0.002, $\eta_{p}^{2}$ = 0.04). Women rated bonding (*p* < 0.001) and control pictures (*p* = 0.01) as more positive than men. Arousal analysis showed a main effect of PICTURE CATEGORY (*F*_(1,260)_ = 100.13, *p* < 0.001, $\eta_{p}^{2}$ = 0.28). No significant differences between sexes were observed for the arousal ratings of bonding and control pictures (main effect of SEX: *F*_(1,260)_ = 2.22, *p =* 0.14, $\eta_{p}^{2}$ = 0.008, interaction between PICTURE CATEGORY and SEX: *F*_(1,260)_ = 0.93, *p* = 0.3, $\eta_{p}^{2}$ = 0.004).

The median score among women on the emotional contagion (empathy) scale was 57.0 ranging from 33 to 71 and among men was 51.0 ranging from 27 to 66. Considering the influence of the empathy trait on valence ratings of bonding and control pictures, the analyses results showed a main effect of PICTURE CATEGORY (*F*_(1,259)_ = 238.42, *p* < 0.001, $\eta_{p}^{2}$ = 0.48), a main effect of EMPATHY (*F*_(1,259)_ = 26.73, *p* < 0.001, $\eta_{p}^{2}$ = 0.09) and an interaction between PICTURE CATEGORY and EMPATHY (*F*_(1,259)_ = 4.78, *p =* 0.03, $\eta_{p}^{2}$ = 0.02). More empathic participants rated bonding pictures as more positive than the less empathic ones (*p* < 0.001). More empathic participants also rated control pictures as more positive than the less empathic ones (*p* < 0.001). Arousal analyses showed a main effect of PICTURE CATEGORY (*F*_(1,259)_ = 119.21, *p* < 0.001, $\eta_{p}^{2}$ = 0.32), a main effect of EMPATHY (*F*_(1,259)_ = 9.92, *p =* 0.001, $\eta_{p}^{2}$ = 0.04) and no significant interaction between PICTURE CATEGORY and EMPATHY (*F*_(1,259)_ = 0.155, *p =* 0.7, $\eta_{p}^{2}$ = 0.0006). Empathy impacted on valence and arousal ratings but do not seem to be specifically associated with the presence of social interaction cues.

Further probing if empathy would also affect ratings of the IAPS categories (unpleasant, neutral, and pleasant) revealed that for valence there was a main effect of PICTURE CATEGORY (*F*_(2,518)_ = 2514,10, *p* < 0.001, $\eta_{p}^{2}$ = 0.91) and of EMPATHY (*F*_(1,259)_ = 9.02, *p* < 0.001, $\eta_{p}^{2}$ = 0.03) and an interaction between PICTURE CATEGORY and EMPATHY (*F*_(2,518)_ = 23.80, *p* < 0.001, $\eta_{p}^{2}$ = 0.084). More empathic participants rated pleasant pictures as more positive and unpleasant pictures as more negative than the less empathic ones (*p* < 0.001 for all comparisons). Arousal analysis showed a main effect of PICTURE CATEGORY (*F*_(2,518)_ = 496.45, *p* < 0.001, $\eta_{p}^{2}$ = 0.66), a main effect of EMPATHY (*F*_(1,259)_ = 7.93, *p* < 0.01, $\eta_{p}^{2}$ = 0.029) and an interaction between PICTURE CATEGORY and EMPATHY (*F*_(2,518)_ = 3.81, *p =* 0.02, $\eta_{p}^{2}$ = 0.01). More empathic participants rated pleasant (*p* = 0.03) and unpleasant (*p* = 0.001) pictures as more arousing than the less empathic ones. Results indicate that empathic individuals are reactive to more diverse emotional cues.

**FIGURE 3:**
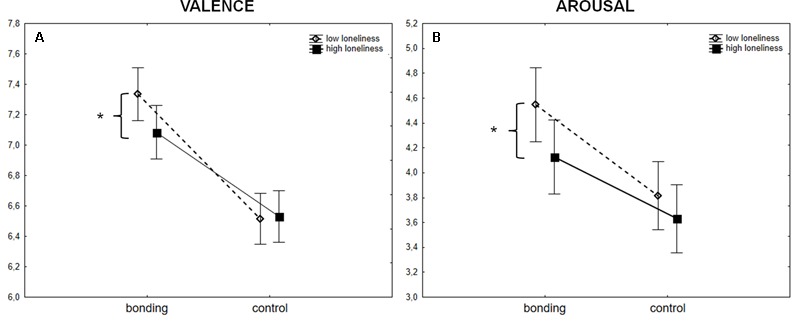
Mean ± standard error of the valence **(A)** and arousal **(B)** ratings of the new set of pictures (bonding and control pictures) for the low loneliness and high loneliness groups. Participants with a score above the median for loneliness are depicted by filled black squares, and those with a score below the median are depicted by empty diamonds. ^∗^*p* = 0.04 for the comparison between low and high loneliness.

The median score among women on the loneliness scale was 34.5 ranging from 21 to 59 and among men was 34.0 ranging from 23 to 55. Considering the influence of the trait of loneliness on valence ratings of bonding and control pictures, results showed a main effect of PICTURE CATEGORY (*F*_(1,261)_ = 241.02, *p* < 0.001, $\eta_{p}^{2}$ = 0.48), an interaction between PICTURE CATEGORY and LONELINESS (*F*_(1,261)_ = 9.05, *p* = 0.003, $\eta_{p}^{2}$ = 0.03) and no significant main effect of LONELINESS (*F*_(1,261)_ = 1.05, *p* = 0.3, $\eta_{p}^{2}$ = 0.004). Lonelier participants rated bonding pictures as less pleasant than less lonely ones (*p* = 0.04). Valence ratings of low and high loneliness sub-groups for control pictures, though, were not significantly different. See **Figure 3A**. Arousal analysis showed a main effect of PICTURE CATEGORY (*F*_(1,261)_ = 121.17, *p* < 0.001, $\eta_{p}^{2}$ = 0.32), an interaction between PICTURE CATEGORY and LONELINESS (*F*_(1,261)_ = 4.44, *p* = 0.04, $\eta_{p}^{2}$ = 0.02) and no significant main effect of LONELINESS (*F*_(1,261)_ = 2.34, *p* = 0.1, $\eta_{p}^{2}$ = 0.009). Lonelier participants rated bonding pictures as less arousing than the less lonely ones (*p* = 0.04). Arousal ratings of low and high loneliness sub-groups for control pictures, though, were not significantly different. See **Figure 3B**. Results point to a specific impact of the presence of social interaction cues on valence and arousal ratings of less lonely individuals compared to the lonelier sub-group.

Checking if trait of loneliness would affect also valence ratings of the IAPS pictures seems to favor the specificity of social interaction scenes. Results showed a main effect of PICTURE CATEGORY (*F*_(2,522)_ = 2301.86, *p* < 0.001, $\eta_{p}^{2}$ = 0.90), but no main effect of LONELINESS (*F*_(1,261)_ = 1.93, *p* = 0.2, $\eta_{p}^{2}$ = 0.007), and no interaction between PICTURE CATEGORY and LONELINESS (*F*_(2,522)_ = 2.62, *p* = 0.07, $\eta_{p}^{2}$ = 0.01). Arousal analysis showed a main effect of PICTURE CATEGORY (*F*_(2,522)_ = 492.33, *p* < 0.001, $\eta_{p}^{2}$ = 0.65), but no main effect of LONELINESS (*F*_(1,261)_ = 0.35, *p* = 0.6, $\eta_{p}^{2}$ = 0.001) and no interaction between PICTURE CATEGORY and LONELINESS (*F*_(2,522)_ = 0.37, *p* = 0.7, $\eta_{p}^{2}$ = 0.001).

## Discussion

The present study successfully created new sets of matched pairs of bonding and control pictures in which social interaction was the main unique contrast between them. Valence and arousal ratings for IAPS pictures correlated with their normative values (Lang et al., 2005). Bonding and control pictures were rated in the pleasant domain. Although both presented a one or two children, bonding pictures were rated more pleasant and arousing than control ones. Being the main contrast between bonding and control pictures, social interaction is, as predicted, an important and relevant appetitive cue.

The influence of sex on valence and arousal ratings for IAPS pictures was already described by Bradley et al. (2001b). Therefore, sex differences were tested only for the new set of pictures. Our results showed that women rated bonding and control pictures as more positive than men. There was no significant difference between women and men in the ratings of arousal for bonding and control pictures.

Social stimuli are widely used in research. Sets of static and dynamic faces (such as schematic and real faces); other social stimuli depicted in photographs, drawings or videos (for a review see, Risko et al., 2012), pictures of dyads of a child and an adult, or a child and a child in scenes of interaction (Campagnoli et al., 2015), and videos of real-life interactions (Muhtadie et al., 2015) have been used in social neuroscience studies. The new sets of pictures tested in the present work were photographs of dyads in which experimental and control pairs were portrayed by the same individuals (one of which being always a child) and each pair was photographed against the same background. The unique feature of the sets is that the social interaction was only present in the “bonding” example of the pair, minimizing other possible influences such as differences in color, complexity, brightness, and contrast between both set of pictures.

A previous study by Campagnoli et al. (2015), using stimuli similar to the present work (except that the dyads of bonding and control pictures were not matched by the same people and not displayed with the same background), demonstrated that the exposure to bonding pictures increased the subjective feelings of sociability, and decreased the feelings of isolation. In respect of psychophysiological measures, the authors also showed that exposure to bonding compared to control pictures increased the electromyographic activity of the fingers flexor muscles during a caress-like movement, while decreasing the amplitude of the motor readiness potential, which was assessed by using the event-related potential technique, derived from the electroencephalogram. According to the authors, these results represent the facilitatory effects of the activation of pre-set cortical motor repertoires evoked by social interaction stimuli, in addition to facilitating the motor output (i.e., greater electromyographic activity). These results indicate that viewing scenes of social interaction prepares an individual’s body for interaction.

More empathic participants rated bonding, control, and pleasant pictures as more positive and unpleasant pictures as more negative than the less empathic ones. Besides, more empathic participants rated pleasant and unpleasant pictures as more arousing than the less empathic ones. Individuals with high scores in the empathy scale were shown to be more likely to respond to different kinds of emotional situations (Balconi and Bortolotti, 2012). In addition, a higher score in empathy was correlated to higher responsiveness to prosocial situations, where more empathetic individuals are more sensitive to external cues that require an empathic response (Balconi and Canavesio, 2013). Moreover, studies show that more empathic individuals are more likely to imitate facial expressions (Sonnby-Borgstrom, 2002a,b; Balconi et al., 2011; Balconi and Bortolotti, 2012). The scale used here to measure the empathy of individuals was related to their experience and exposure to emotional expressions (Doherty, 1997). As empathy acts as a social facilitator of processes for detection of facial emotions, more empathic individuals are consequently more skilled in processing facial expressions (Balconi and Canavesio, 2016). In this respect, it is reasonable to suppose that the increase in valence and arousal ratings of emotional pictures among more empathic individuals compared to less empathic ones can be explained by their greater capacity to be involve in emotional stimuli in general.

Finally, our study showed that more lonely participants specifically rated bonding pictures as less pleasant and less arousing than the less lonely individuals. Cacioppo and Hawkley (2009) demonstrated that the social isolation trait is correlated with decreased pleasure in social interactions. It was suggested that individuals who have higher scores in loneliness are less sensitive to detecting visual social cues (Kanai et al., 2012). Therefore, we can suggest that the reduced emotional response to social interaction stimuli among lonelier individuals is associated with a less pleasant response, and a reduced arousal reaction to these stimuli. A study found that lonely people are less likely to expect good social interactions and, at the same time, their motivations are consistent with avoiding social stimuli (Gable, 2006). This could explain the lower pleasantness elicited by bonding pictures in lonelier individuals.

A number of caveats need to be noted regarding the present study: (i) the sample comprised only undergraduate students; (ii) the sample was comprised mostly by women (64%), and (iii) other questionnaires to assess distinct personality traits (e.g., depression or anxiety) could be used. Future studies should focus on the investigation of the self-reported emotional ratings of this new set of bonding and matched-control pictures using other samples, for instance, children, elderly and psychiatric patients, etc. Importantly, investigating peripheral and central psychophysiological activity during passive viewing pictures paradigms or while performing tasks is needed. A possible interesting approach could be to use this new set of pictures as a therapy for loneliness, which impact so negatively in physical and mental health.

We conclude that the new set of social bonding and control pictures of children and adults, selectively matched by the same dyads and the same background scenes and comprising direct social interaction (bonding) or non-direct social interaction (control), have reliable valence and arousal ratings, assessed accordingly to the methodology proposed by Lang et al. (2005). This was confirmed by the similarity of the valence and arousal ratings of the IAPS pictures when compared to the present sample. In addition, bonding pictures were rated more pleasant and more arousing than control pictures, showing the importance of social interaction for human beings. Sex influenced the assessment of valence of the new set of pictures. Regarding individual emotional traits, empathy modulated the valence of all pictures except by neutral ones and the arousal of pleasant and unpleasant pictures. Loneliness, in turn, affected only the ratings of bonding pictures, showing the specificity of this emotional trait over emotional ratings of social interaction scenes.

## Author Contributions

RC, IM, VR-R, EV, and GS designed research; HS, BM, and CA performed the experiments; HS, BM, CA, GS, and RÁ analyzed data; RC, IM, VR-R, EV, GS, and RÁ participated in data interpretation; HS, BM, and GS wrote the paper. All the authors critically reviewed and approved the final version of the manuscript.

## Conflict of Interest Statement

The authors declare that the research was conducted in the absence of any commercial or financial relationships that could be construed as a potential conflict of interest.

## Footnotes

1 The following IAPS pictures were employed: UNPLEASANT PICTURES: Mutilations – 3060, 3080, 3110, 3130; Pollution – 9110, 9230, 9270; Loss – 2900, 9220, 9421; Illness – 2205, 3230, 3350; Contamination – 9320, 7380, 9300, 9830; Accidents – 9050, 9600, 9920, 9911; Violence – 3530, 6260, 6350, 3180, 9120; Animal and human attack – 1300, 1120, 1930, 6510. NEUTRAL PICTURES: Objects – 7235, 7020, 7025, 7040, 7010, 7006, 7034, 7031, 7009, 7080, 7035, 7175, 7050, 7150, 7000, 7090, 7100, 7130, 7190, 7170, 7233, 7205, 7211, 7234, 7224, 7187; Mushrooms – 5500, 5535,5533, 5530. PLEASANT PICTURES: Erotic couples – 4690, 4650, 4660, 4659; Food – 7400, 7350, 7470, 7480, 7330; Sports and adventure – 8470, 8170, 8180, 8490; Nature – 5780, 5849, 5760.

2 The following IAPS pictures were employed in the practice task: 7002, 5200, 3064, 7450, 6570, 7060, 9102, 7185, 2057.
